# Autoimmune Diseases of Digestive Organs—A Multidisciplinary Challenge: A Focus on Hepatopancreatobiliary Manifestation

**DOI:** 10.3390/jcm10245796

**Published:** 2021-12-11

**Authors:** Lumir Kunovsky, Petr Dite, Petr Jabandziev, Zdenek Kala, Jitka Vaculova, Tomas Andrasina, Matej Hrunka, Martina Bojkova, Jan Trna

**Affiliations:** 1 Department of Gastroenterology and Internal Medicine, University Hospital Brno, 625 00 Brno, Czech Republic; kunovsky.lumir@fnbrno.cz (L.K.); pdite.epc@gmail.com (P.D.); vaculova.jitka@fnbrno.cz (J.V.); jan.trna@seznam.cz (J.T.); 2 Department of Surgery, University Hospital Brno, 625 00 Brno, Czech Republic; kala.zdenek@fnbrno.cz; 3 Faculty of Medicine, Masaryk University, 625 00 Brno, Czech Republic; andrasina.tomas@fnbrno.cz (T.A.); hrunka.matej@fnbrno.cz (M.H.); 4 Department of Gastroenterology and Internal Medicine, University Hospital Ostrava, 728 00 Ostrava, Czech Republic; martina.jelsikova@seznam.cz; 5 Faculty of Medicine, University of Ostrava, 703 00 Ostrava, Czech Republic; 6 Department of Pediatrics, University Hospital Brno, 613 00 Brno, Czech Republic; 7 Central European Institute of Technology, Masaryk University, 625 00 Brno, Czech Republic; 8 Department of Radiology and Nuclear Medicine, University Hospital Brno, 625 00 Brno, Czech Republic; 9 Department of Gastroenterology and Digestive Endoscopy, Masaryk Memorial Cancer Institute, 602 00 Brno, Czech Republic

**Keywords:** human leukocyte antigen, celiac disease, inflammatory bowel disease, autoimmune hepatitis, primary sclerosing cholangitis, primary biliary cholangitis, autoimmune pancreatitis, IgG4-related sclerosing cholangitis, IgG4-related hepatopathy

## Abstract

It is well known that some pathological conditions, especially of autoimmune etiology, are associated with the HLA (human leukocyte antigen) phenotype. Among these diseases, we include celiac disease, inflammatory bowel disease, autoimmune enteropathy, autoimmune hepatitis, primary sclerosing cholangitis and primary biliary cholangitis. Immunoglobulin G4-related diseases (IgG4-related diseases) constitute a second group of autoimmune gastrointestinal, hepatobiliary and pancreatic illnesses. IgG4-related diseases are systemic and rare autoimmune illnesses. They often are connected with chronic inflammation and fibrotic reaction that can occur in any organ of the body. The most typical feature of these diseases is a mononuclear infiltrate with IgG4-positive plasma cells and self-sustaining inflammatory response. In this review, we focus especially upon the hepatopancreatobiliary system, autoimmune pancreatitis and IgG4-related sclerosing cholangitis. The cooperation of the gastroenterologist, radiologist, surgeon and histopathologist is crucial for establishing correct diagnoses and appropriate treatment, especially in IgG4 hepatopancreatobiliary diseases.

## 1. Introduction

Autoimmune diseases of digestive organs can be divided into three groups: (1) autoimmune digestive diseases associated with human leukocyte antigen (HLA), (2) immunoglobulin G4-related diseases (IgG4-RD) and (3) other autoimmune gastrointestinal diseases. It is well known that some pathological conditions, especially of autoimmune etiology, are associated with different HLA phenotypes [1,2]. Among these diseases, we include celiac disease, inflammatory bowel disease, autoimmune hepatitis, primary sclerosing cholangitis and primary biliary cholangitis. It is widely recognized that incidence of autoimmune diseases is generally increasing. We can demonstrate this in the incidence of autoimmune hepatitis (AIH). In an English study published by Grønbaek et al., the incidence of AIH grew between the years 1997 and 2015 from 1.27 to 2.56 per 100,000 population per year [3]. Very similar data were reported for a Danish cohort, in which case the incidence doubled between 1994 and 2012 [4]. This demonstrates the importance of our topic.

## 2. Autoimmune Digestive System Diseases Associated with HLA

### 2.1. Celiac Disease

Celiac disease is represented by a gluten-sensitive enteropathy. It develops in genetically susceptible individuals. The main role is played by T cell lymphocytes reactivity against gluten [5]. The diagnostic criteria are well defined in children; the last ESPGHAN (European Society for Pediatric Gastroenterology, Hepatology and Nutrition) guidelines admit the non-biopsy diagnostic approach, based only on serological tests (IgA antibodies against transglutaminase 2, IgA endomysial antibodies and total IgA antibodies). In defined cases, it is necessary to perform duodenal biopsy [6]. However, in adults the biopsy is necessary for the diagnosis of celiac disease [7]. Treatment is based upon a gluten-free diet. Celiac disease was first reported to be associated with HLA class 1 molecule B8. Later, association was shown with four haplotypes (celiac dimers) [8]. Celiac disease is an autoimmune illness and is today an important candidate for the clinical use of human leukocyte antigen isotype DQ (HLA-DQ) genotyping. The main determinants for genetic susceptibility are HLA-DQA1 and HLA-DQB1 genes encoding for HLA-DQ2 and HLA-DQ8 molecules [9]. In this context, HLA analysis seems to be an important resource in the diagnostic armamentarium for serving a population at high risk of celiac disease [10]. According to recent guidelines, HLA testing attained a new role in the diagnostic approach, because of its high negative predictive value [6]. Very interesting is the presence of autoantibodies against Saccharomyces cerevisiae (ASCA) in celiac patients, mainly before initiating a gluten-free diet. These autoantibodies are more specific for Crohn’s disease. Granito et al. [11] reported, in their study, 59% of celiac patients having ASCA positivity. The potential explanation of these phenomenon is the immune response for small bowel inflammation [11,12].

### 2.2. Inflammatory Bowel Disease (IBD)

The role of HLA in patients with inflammatory bowel disease (IBD) remains uncertain. In addition, unclear is the etiology of IBD, which is assumed to include a combination of an individual’s genetic background, alteration of gut microbiota, immune dysregulation and environmental factors [13]. HLA typing might be useful in discriminating Crohn’s disease (CD) and ulcerative colitis (UC) [14,15] and its application may improve the sensitivity and specificity of serological markers. However, HLA typing is not routinely used in IBD patients. In clinical practice, we mainly use two serological markers of IBD, which are perinuclear anti-neutrophil cytoplasmic autoantibodies (pANCAs) and anti-Saccharomyces cerevisiae antibodies (ASCAs). ASCA, we mainly found in serum of CD patients, with sensitivity at 37–72% and specificity at 72–100%. ANCA are more common for UC patients with sensitivity reaching 70% and specificity almost 98% in some studies [16]. In a study by Bouzid et al. [17], patients with IBD showed significantly increased frequency of the homozygous DR Beta 1 (DRB1) 07 genotype [17].

The HLA system is also considered to be a major genetic marker and is associated with extraintestinal manifestation of IBD. For example, HLA-B27-positive patients with IBD have higher risk for developing ankylosing spondylitis. Primary sclerosing cholangitis, another autoimmune disease which is often coexisting with IBD, has also been associated with various HLA alleles. Generally, patients with IBD have increased an risk of various autoimmune and inflammatory diseases [18]. Treatment of IBD is a typical example of a multidisciplinary approach. The patient could be treated by aminosalicylates, corticosteroids, immunosuppressant, biologics or by surgery. Diet plays an especially important role in treating pediatric patients [19,20].

IBD is a diagnosis which offers wide scope for use of different biological drugs. They interfere with immune system on different levels. For example, in cytokine production, signaling pathway in T cell activation or inhibiting Januse kinase [21,22]. The most commonly used are Tumor Necrosis Factor alfa antibodies (antiTNF-alfa) such as infliximab or adalimumab. These biologics have also been tried in the treatment of refractory autoimmune hepatitis [23] or primary sclerosing cholangitis [24]. However, antiTNF-alfa is currently not used in the treatment of autoimmune hepatitis, nor in primary sclerosing cholangitis. Despite the promise of biologics being able to target specific cellular and humoral pathways, results have been generally poor and safety has not been as expected [25].

We also have possibility to use some novel biologics for IBD treatment with promising therapeutic effects such as vedolizumab or ustekinumab [26,27].

### 2.3. Autoimmune Enteropathy (AIE)

Autoimmune enteropathy (AIE) in adults is a heterogeneous disease associated with a variety of circulating gut antibodies and predisposition to autoimmunity [28].

The pathophysiology of AIE is not exactly known. Dysfunction of CD25^+^CD4^+^ regulatory T cells probably plays an important role [29]. AIE is a result of humoral immune response involving anti-enterocyte antibodies (which have been detected in a majority of those affected) and anti-goblet cell antibodies [30]. Anti-enterocyte antibodies are not specific for AIE, as they have been described in such other diseases as allergic enteropathy, HIV infection and IBD [31]. On the other hand, other autoantibodies in patients with AIE are presented (e.g., antinuclear antibody or anti-smooth muscle antibodies) [32]. Antibodies against villin, a protein occurring in intestinal microvilli and proximal renal tubules, can be used in the diagnosis of immunodysregulation polyendocrinopathy enteropathy X-linked syndrome [33].

AIE is a rare condition, clinically connected with refractory diarrhea and malnutrition, mainly in children. Other typical changes are histological changes in small intestinal biopsy. In many patients, immunosuppressive therapies are principally used. Diagnostic criteria are detailed in Table 1 (adapted and modified from [28]).

Criteria 1–4 are required for a definite diagnosis of AIE. Presence of anti-enterocyte antibodies is an important diagnostic support, but their absence does not exclude the diagnosis of AIE. In the light of study from Biagi et al., we dare to remove anti-goblet cell antibodies from previous proposed diagnostic criteria. They are more unspecific than expected [34].

### 2.4. Autoimmune Hepatitis (AIH)

Autoimmune hepatitis (AIH) is a non-resolving inflammation of the liver. The disease reflects a complex interaction between triggering factors, autoantigens, genetic predisposition and immunoregulatory networks [35]. The disease usually is discriminated into three subtypes. Type 1 AIH is associated with antinuclear antibodies (ANAs) and/or smooth muscle antibodies (SMA). Type 2 AIH affects mostly children. Typical is a positivity of antibodies to liver-kidney microsome type 1 (LKM-1). Type 3 AIH is associated with soluble liver antigens. Diagnostic criteria for AIH are presented in Table 2.

AIH is a global disease, occurring most frequently as type 1. HLA typing was part of original and revised diagnostic criteria of AIH [37], but now in simplified criteria is not accepted as a routine test for AIH, although it probably could be useful in distinguishing overlapping syndromes, in differentiating various types of autoimmune liver disease [38] or explaining regional differences in incidence of the disease [39]. Treatment of AIH includes using corticosteroids in induction therapy and azathioprine as a first-line maintenance treatment [40].

### 2.5. Primary Biliary Cholangitis (PBC)

Formerly known as primary biliary cirrhosis, primary biliary cholangitis (PBC) is a chronic cholestatic disease of unknown etiology and affecting mainly females. Essential to positive diagnosis is a combination of serological markers of cholestasis, the presence of autoantibodies such as antimitochondrial antibodies (AMA) and PBC specific antinuclear antibodies (ANA) anti-gp210 and anti-sp100 and imaging of liver and bile ducts. In unclear cases, we can perform liver biopsy [41]. The disease is characterized by the slow, progressive destruction of small intrahepatic bile ducts and by autoantibodies positivity. Ursodeoxycholic acid and obeticholic acid are available for managing PBC [41]. The end-point of this disease is liver cirrhosis and liver failure. For end-stage liver disease, liver transplant is the method of choice. The pathogenesis is multifactorial; genetic and environmental factors can induce an autoimmune reaction against bile ducts. The disease is strongly associated with several HLA haplotypes. According to a Scandinavian study, the most prominent risk HLA haplotypes are HLA-DRB1*13:01-DQA1*01:03-DQB1*06:03 [42].

Genetic factors are very important and documented by the high concordance rate of PBC among monozygotic twins [43]. This group of autoimmune gastrointestinal and hepatobiliary diseases is characterized not only by positivity of autoantigens, but also by positivity of HLA markers, genetic association and cytotoxic T cells population.

### 2.6. Primary Sclerosing Cholangitis (PSC)

Primary sclerosing cholangitis (PSC) is a rare, chronic cholestatic liver disease that can lead to liver fibrosis and cirrhosis. Pathologically, PSC is characterized by an inflammation and destruction of extra- and intrahepatic bile ducts. The etiology of this disease is unknown. Association between PSC and IBD has been described [44]. IBD was found in 80% of all patients with PSC, but PSC was found in just 5% of all IBD patients [45].

The pathogenetic mechanism is still unknown. Inflammatory changes are centered on the biliary epithelium and damage of the biliary tree is frequently observed. PSC is probably an immunologically mediated process with HLA association [46]. Experimental studies have shown that bacterial overgrowth also plays a role and there is a link with ulcerative colitis and gene mutations (e.g., *CFTR* mutation (cystic fibrosis transmembrane receptor mutation)) [47,48]. Subtypes of PSC are large-duct PSC, small-duct PSC, overlap syndrome with AIH and PSC with elevated IgG4 in serum and/or tissue [49]. Imaging methods and estimation of serum alkaline phosphatase level play crucial roles in diagnosis. Liver biopsy is usually unnecessary, although this could be helpful in differential diagnosis (e.g., to determine AIH overlap syndrome or small-duct PSC). The prognosis for this disease varies and the course of the disease is connected with serum alkaline phosphatase level.

There is no effective pharmacological therapy. It remains unclear whether or not administering ursodeoxycholic acid can be effective. Liver transplantation is an effective treatment for end-stage liver disease [50].

## 3. Immunoglobulin G4-Related Diseases (IgG4-RD)

The second group of autoimmune gastrointestinal, hepatobiliary and pancreatic diseases is termed immunoglobulin G4-related diseases (IgG4-RD). IgG4-RD is a group of systemic and rare autoimmune diseases, often connected with chronic inflammation and fibrotic reaction that can occur in any organ of the body [51]. Etiology of IgG4-RD remains unclear. Current knowledge suggests that IgG4-RD are autoimmune disorders, where T and B cell lymphocytes are involved in pathophysiology [52]. The role of IgG4 antibodies has two explanations. The first is that IgG4 destroys tissues. The second is that high levels of IgG4 may reflect only overexpression of antibodies as a response to unknown inflammatory stimulus [53]. The most typical feature of these diseases is a mononuclear infiltrate with IgG4-positive plasma cells and self-sustaining inflammatory response.

IgG4 is an immunoglobulins fraction accounting for just 5.0% of the IgG pool. IgG4 is physiologically produced after a long-term exposure to food or environmental antigens. A current hypothesis is that the transformation from B cells to plasma cells and activation of eosinophilic granulocytes can probably be triggered by an initial Th1-type immune response via secretion of proinflammatory cytokines [36].

It is important that the level of plasmablast correlates with the diagnosis and disease activity much better than it does with serum IgG4 level. Plasmablast seems to be a precursor of tissue-resident antibody-producing plasma cells [54]. Oligoclonal cytotoxic T cell populations, such as CD4-positive cytotoxic T lymphocytes, correlate with disease activity and therefore may be better indicators of IgG4-RD activity, since serum IgG4 levels are not always increased. The cytotoxic T lymphocytes probably serve as vital antigen-presenting cells to rogue T cells, hence perpetuating the inflammation via secretion of profibrotic cytokines—growth factor-beta 1 and interleukin-1 beta—leading to chronic inflammation and fibrosis. A new biomarker consists in quantification of plasmablasts in peripheral blood. It has shown strong sensitivity and specificity for diagnosis of IgG4-RD [55]. Diagnostic criteria for IgG4-RD target organs are presented in Table 3.

### 3.1. Autoimmune Pancreatitis (AIP)

The most frequently encountered manifestation of IgG4-RD in the gastrointestinal system is autoimmune pancreatitis (AIP). Two subtypes of AIP have been described. Type 1 AIP is a typical pancreatic manifestation of IgG4-RD, while type 2 AIP is an autoimmune disease of the pancreas.

Type 1 AIP is known as lymphoplasmacytic sclerosing pancreatitis, or LPSP. There exists an international diagnostic consensus that can be followed when making the diagnosis according to the major IgG4-RD criteria. This consensus was published by Shimosegawa et al. [57] in 2011. Type 1 AIP is a rare disease, but in a German retrospective cross-sectional analysis the prevalence of AIP was 9.1%. All patients were nonalcoholic [58]. While type 2 AIP shares several features with type 1, a low amount or absence of IgG4 plasma cell infiltration and a presence of granulocytic epithelial lesions (GEL) are the most important diagnostic markers for type 2 AIP. Moreover, elevated serum IgG4 in type 2 AIP is very rare [59]. Diagnostic criteria for types 1 and type 2 AIP can be seen in Table 4 and Table 5, respectively (adapted from [57]). Characteristics of types 1 and 2 AIP are summarized in Table 6 (adapted from [60]).

Despite international guidelines for diagnosing AIP, its differentiation from pancreatic cancer is still challenging [61]. In an interesting paper, Shih et al. report finding that patients with pancreatic cancer had significantly different profiling of IgG-glycosylation than did patients with AIP [62]. IgG glycosylation could probably be a useful marker in differentiating with high accuracy (sensitivity 94.6%, specificity 92.9%) between pancreatic ductal adenocarcinoma and focal form of AIP.

### 3.2. IgG4-Related Hepatobiliary Disease

The most common IgG4-RD among hepatobiliary diseases is IgG4-related sclerosing cholangitis. This disease is often associated with other organ manifestations of IgG4-related illnesses, most typically with type 1 AIP [63]. The disease is completely reversible under glucocorticoid therapy, which is typical for IgG4-RD. The most typical clinical symptoms in diagnosing IgG4-related sclerosing cholangitis are painless jaundice, pruritus, abdominal discomfort and oftentimes association with diabetes mellitus [64]. Biochemical markers of cholestasis are positive, the level of CA 19-9 could be very high (albeit with positive response to steroid therapy), serum IgG4 is elevated to >3 times the upper limit [65]. Histopathological criteria for IgG4-related sclerosing cholangitis are similar to those for AIP and are presented in Table 7.

**Figure 1:**
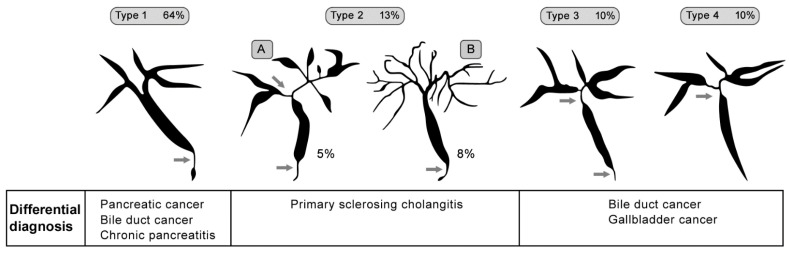
Classification of IgG4-related sclerosing cholangitis (prepared in accordance with Nakazawa et al. [67] and created in collaboration with the Service Center for E-Learning at Masaryk University, Faculty of Informatics).

In cholangiography, four typical images have been described and are classified by Nakazawa et al. [67]. The typical features are strictures of the lower part of the common bile duct, intrahepatic segmental or diffuse stricture, or a combination of hilar stricture with the lower part of common bile duct-related hepatopathy strictures. Figure 1 depicts IgG4-related sclerosing cholangitis classification with summary differential diagnosis (prepared in accordance with Nakazawa et al. [67]).

**Figure 2:**
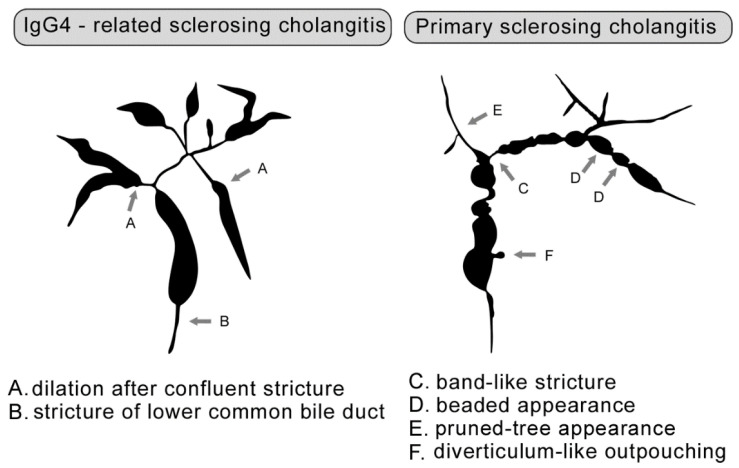
Comparison of cholangiographic findings of IgG4-related sclerosing cholangitis and PSC (prepared in accordance with Ohara et al. [68] and created in collaboration with the Service Center for E-Learning at Masaryk University, Faculty of Informatics).

In differential diagnoses, we must also always consider PSC. Figure 2 provides a comparison of the cholangiographic findings of IgG4-related sclerosing cholangitis and PSC (prepared in accordance with Ohara et al. [68]).

In IgG4-related hepatopathy, five manifestations of liver involvement have been identified: (1) portal inflammation, (2) lobular hepatitis, (3) portal sclerosis, (4) lobular cholestasis and (5) bile duct obstruction [56]. The important diagnostic feature for IgG4-related hepatopathy is increasing IgG4 plasma cells. Nevertheless, the number of case reports on this topic is limited and the clinical relevance of IgG4-related AIH remains unclear. Another problem is that different manifestations of hepatic changes in IgG4-RD have been reported in very small and retrospective studies [69,70].

### 3.3. Other IgG4-Related Gastrointestinal Diseases

Only a few clinical papers have been published about IgG4-related gastrointestinal diseases of the esophagus, stomach and bowel, with diffuse infiltration of the gastric mucosa by IgG4 + plasma cells being the most commonly described [71]. This infiltration does not fulfill the other histopathological criteria typical for IgG4-RD, but has been shown to disappear after oral therapy with steroids. In some patients, thickened (up to 15 mm) and nodular gastric mucosa can be seen [72].

In IgG4-related gastric lesions, focal polypoid lesions or focal masses up to 3 cm, gastric ulcer, diffuse thickening of the wall and association with local lymphadenopathies have been observed [73].

In colon biopsies from a set of 119 patients with IBD, Topal et al. found IgG4 positivity in 17.6% [74]. Obiorah et al. confirmed the diagnosis of IgG4-related esophagitis in 8 out of 18 patients [75]. Sporadic cases have also been described in the small bowel or rectum [76,77].

## 4. Other Autoimmune Gastrointestinal Diseases

### Autoimmune Gastritis (AIG)

Autoimmune gastritis (AIG) is an immune-mediated chronic disease with mostly mild or non-specific clinical manifestation. It affects corpuscular acid-producing mucosa, especially parietal and chief cells, and leads to intrinsic factor deficiency and hypo- or achlorhydria [78]. AIG’s etiology, which is not yet fully clarified, is marked by an important influence of genetic, hormonal and environmental factors in combination with immune dysregulation [79]. Although AIG is often reported to be a silent disease, we can encounter with it nonspecific gastrointestinal symptoms such as dyspepsia, postprandial fullness, nausea or early satiety. The most typical symptom is anemia, mainly from iron deficiency or as a pernicious anemia from vitamin B_12_ deficiency [80]. Essential to its diagnosis is upper gastrointestinal endoscopy with histological assessment of gastric biopsies. Anti-parietal cell antibodies provide a useful marker [78]. Treatment options for this condition are substantially limited and mainly focused on micronutrient supplementation. No anti-inflammatory, immunosuppressive or biological therapy is available [81].

## 5. Conclusions

Autoimmune diseases of the gastrointestinal organs constitute a huge gastroenterological challenge. Many gastrointestinal organs undergo biochemical and histopathological changes in connection with IgG4-RD or the HLA system. Diagnosis and therapy of these diseases require a multidisciplinary approach and cooperation among gastroenterologists, hepatologists, surgeons, immunologists, histopathologists and radiologists. There can be no doubt that autoimmune organ disorders have a place in the broad and multidisciplinary field of gastroenterology.

## Figures and Tables

**Table 1:** Proposed diagnostic criteria for adult autoimmune enteropathy (created in accordance with Akram et al. [28]).

1. Adult-onset chronic diarrhea (>6 weeks duration)
2. Malabsorption
3. Specific small bowel histology: a. Partial/complete villous blunting b. Deep crypt lymphocytosis c. Increased crypt apoptotic bodies d. Minimal intra-epithelial lymphocytosis
4. Exclusion of other causes of villous atrophy, including celiac disease, refractorysprue and intestinal lymphoma
5. Anti-enterocyte antibodies

**Table 2:** Simplified diagnostic criteria for AIH (created in accordance with Hennes et al. [36]).

Criteria	Cut-off	Points
ANA or SMA	≥1:40	1
ANA or SMA	≥1:80	
or LKM	≥1:40	2 (max. 2 points for all antibodies)
or SLA	Positive	
IgG	>Upper normal limit	1
	>1.10 times upper normal limit	2
Liver histology (evidence of hepatitis is a necessary condition)	Compatible with AIH Typical AIH	1 2
Absence of viral hepatitis	Yes	2 ≥6: probable AIH ≥7: definite AIH

**Table 3:** Diagnostic criteria for IgG4-RD target organs (created in accordance with Backhus and Löhr et al. [51,56]).

1. Clinical examination
a. Organ swelling
b. Pseudotumor
c. Jaundice
d. Minimal intra-epithelial lymphocytosis
2. Imaging a. Diffuse or localized organ swelling b. Pseudotumor c. Pancreatic rim (in case of pancreatic involvement) d. “Sausage-like” pancreas (in case of pancreatic involvement)
3. Assessing serum IgG4 concentration (upper level of normal = 135 mg/dL, but only levels higher than 4× the upper level seems to have clear diagnostic value) 4. Presence of 3 major histopathological characteristics a. Lymphoplasmacellular infiltrate with IgG4+ plasma cells (ca 100%) b. Storiform fibrosis (ca 75%) c. Obliterative phlebitis (ca 45%) (see also Table 4)

**Table 4:** Simplified international diagnostic criteria for type 1 autoimmune pancreatitis (created in accordance with Shimosegawa et al. [57]).

Criteria	Description
Pancreas histology (H)	Lymphoplasmacytic sclerosing pancreatitis (LPSP, core biopsy/resection). At least 3 of the following: periductal lymphoplasmacytic infiltrate without granulocytic infiltration, obliterative phlebitis, storiform fibrosis, abundant (>10 cells/high-power field) IgG4-positive cells
Parenchymal imaging (P)	Typical: diffuse enlargement with delayed enhancement (sometimes associated with ring-like enhancement)
Ductal imaging (D)	Long (>1/3 length of the main pancreatic duct) or multiple strictures without marked upstream dilation
Serology (S)	IgG4 > 2× upper normal limit
Other organ involvement (OOI)	1 or 2 Histology of extrapancreatic organs: Lymphoplasmacytic infiltration with fibrosis and without granulocytic infiltration Storiform fibrosis Obliterative phlebitis IgG4-positive plasma cells Typical radiological evidence Segmental/multiple proximal bile duct stricture Retroperitoneal fibrosis
Response to steroid therapy (Rt)	Rapid (≤2 weeks) radiologically demonstrable resolution or marked improvement in pancreatic/extrapancreatic manifestations

**Table 5:** Simplified international diagnostic criteria for type 2 autoimmune pancreatitis (created in accordance with Shimosegawa et al. [57]).

Criteria	Description
Histology (H)	Idiopathic duct centric pancreatitis (IDCP): Both of the following: Granulocytic infiltration of duct wall (GEL) with or without granulocytic acinar inflammation Absent or scant (0–10 cells/high-power field) IgG4-positive cells
Parenchymal imaging (P)	Typical: diffuse enlargement with delayed enhancement (sometimes associated with rim-like enhancement)
Ductal imaging (D)	Long (>1/3 length of the main pancreatic duct) or multiple strictures without marked upstream dilatation
Other organ involvement (OOI)	Clinically diagnosed inflammatory bowel disease
Response to steroid therapy (Rt)	Rapid (≤2 weeks) radiologically demonstrable resolution or marked improvement in pancreatic manifestations

**Table 6:** Characteristics of and fundamental differences between type 1 and type 2 autoimmune pancreatitis (created in accordance with Webster et al. [60]).

	Type 1 (LPSP)	Type 2 (IDCP)
IgG4-RD	Yes	No
Prevalence	Asia > USA/Europe	USA/Europe > Asia
Sex	M > F	M = F
Worldwide percentage (%)	>90	<10
Age predominance (years)	>50	30–50
Initial icterus (%)	>60	<30
Acute abdominal pain (%)	<30	>60
Elevated serum IgG4 (%)	>70	<10
Histopathology	Storiform fibrosis, LPSP, obliterative phlebitis	IDCP, GEL
Affection of other organs	Yes	No
Association with IBD (%)	<10	>40
Steroid response (%)	>90	>90
Relapse after steroid therapy (%)	>40	<10

AIP—autoimmune pancreatitis; IgG4-RD—IgG4-related disease; LPSP—lymphoplasmacytic sclerosing pancreatitis; IDCP—idiopathic duct centric pancreatitis; GEL—granulocytic epithelial lesions; IBD—Inflammatory bowel disease.

**Table 7:** Histopathological criteria for IgG4-related sclerosing cholangitis (created in accordance with Deshpande et al. [66]).

Obliterative phlebitis Storiform fibrosis Lymphoplasmacellular infiltrate with more than 10 IgG4+ plasma cells per high- power field

## Data Availability

None.
